# Soil Nitrogen and Flooding Intensity Determine the Trade-Off between Leaf and Root Traits of Riparian Plant Species

**DOI:** 10.3390/plants13070978

**Published:** 2024-03-29

**Authors:** Hang Zou, Wanyu Wang, Jinxia Huang, Xiaohong Li, Maohua Ma, Shengjun Wu, Cunfeng Zhao

**Affiliations:** 1 The College of River and Ocean Engineering, Chongqing Jiaotong University, Chongqing 400074, China; zouhang@cigit.ac.cn (H.Z.); huangjinxia@cigit.ac.cn (J.H.); 2 Chongqing Institute of Green Intelligent Technology, Chinese Academy of Sciences, Chongqing 400714, China; wangwanyu21@mails.ucas.ac.cn (W.W.); lixiaohong19@mails.ucas.ac.cn (X.L.); wsj@cigit.ac.cn (S.W.); zhaocunfeng@cigit.ac.cn (C.Z.); 3 Chongqing College, University of Chinese Academy of Sciences, Chongqing 400714, China

**Keywords:** leaf traits, root traits, trade-offs, flooding intensity, soil nitrogen, adaptive strategy, riparian communities, life history

## Abstract

The investigation into trade-offs among plant functional traits sheds light on how plants strategically balance growth and survival when facing environmental stress. This study sought to evaluate whether trade-offs observed at both community and individual species levels could indicate adaptive fitness across an intensity of flooding intensity. The study was conducted at 25 sampling sites spanning approximately 600 km along the riparian zone in the Three Gorges Reservoir area, China. The findings revealed that, along the flooding gradient, the overall riparian community did not exhibit significant trade-offs between leaf and root traits. Examining three broadly distributed dominant species (*Cynodon dactylon*, *Xanthium strumarium*, and *Abutilon theophrasti*), perennial plants showed pronounced trade-offs under low flooding intensity, while annuals exhibited trade-offs under moderate and low flooding intensity. The trade-offs were evident in traits related to nitrogen-carbon resources, such as specific leaf area, root tissue density, and photosynthetic rate. However, under strong flooding intensity, the relationship between leaf and root traits of the species studied was decoupled. Furthermore, the study identified a significant correlation between soil nitrogen and the trade-off traits under moderate and low flooding intensity. Integrating results from the CSR (Competitors, Stress-tolerators, Ruderals) strategy model, species niche breath analysis, and nitrogen-regulated trade-off, the study revealed that, in the face of high flooding intensity, perennial species (*C. dactylon*) adopts an S-strategy, demonstrating tolerance through a conservative resource allocation that decouples leaf-root coordination. Annual species (*X. strumarium* and *A. theophrasti*), on the other hand, exhibit niche specialization along the flooding gradient, employing distinct strategies (R- and C-strategy). As flooding stress diminishes and soil nitrogen level decreases, plant strategies tend to shift towards an R-strategy with a competition for reduced N resources. In conclusion, the study highlighted the pivotal roles of soil nitrogen and flooding intensity acting as the dual determinants of species growth and tolerance. These dynamics of growth-tolerance balance were evident in the diverse trade-offs between leaf and root traits of individual plant species with different life histories, underscoring the array of adaptive strategies employed by riparian plants across the flooding intensity gradient.

## 1. Introduction

Plants, in response to environmental changes, have developed adaptive strategies characterized by a spectrum of internal physiological and external morphological traits. These traits, reflective of growth, development, and reproduction, play a crucial role in how plants respond to external environmental shifts [1]. Individually or collectively, these traits serve as indicators of the responses of plant individuals, populations, and communities to changes in their environment. The survival of plants is contingent upon possessing specific traits that are well-suited to their habitat [2,3,4]. The CSR (Competitors, Stress-tolerators, Ruderals) strategy model, proposed by Grime [5], is widely employed to assess transformations in plant communities induced by environmental stress. Consequently, studies focused on plant-environment relationships through a trait-based approach offer insights into the adaptive strategies of plants to environmental changes. These investigations serve as a foundation for understanding species fitness, particularly in the context of the survival-growth trade-off [6]. Furthermore, such studies can contribute to the broader understanding of ecosystem processes and associated functions [7].

In the quest to discern the plant strategy balancing survival and growth, the examination of plant trait plasticity extends beyond the confines of individual or grouped traits. Instead, there is a growing emphasis on exploring intrinsic relationships, particularly the trade-offs between plant traits [8]. The trait phenotypic plasticity depends on resource trade-off strategies at the phenotypic organ level [9], which vary along an environmental gradient. This resource-based trade-off reflected by traits is known as the ‘plant economy spectrum’ [10]. It describes a class of important plant traits that have emerged during long-term evolution based on the trade-offs between resource investments and returns. These traits and their trade-offs are important manifestations of plant phenotype plasticity, representing survival strategies among different plant species [11].

The economic spectrum, initially proposed for plant leaf traits, links these traits to plant strategies [12]. The leaf, vital in the food chain, is a key plant organ for energy synthesis. Global studies reveal diversity in leaf structure and function, with the leaf economic spectrum distinguishing between fast-growing species that acquire resources rapidly and slow-growing species that conserve resources [13]. Expanding this, the spectrum is applied to plant root systems [14]. Roots, also vital for ecosystems, supply nutrients and facilitate carbon transfer. Resource-acquisitive species have high specific root length, high root nitrogen, and low root tissue density, while resource-conservative species have thicker and denser roots [10,15,16]. However, the allocation of resources in response to environmental factors and the coordination of ecosystem responses remain subject to ongoing investigation. Furthermore, in studying Mediterranean woody plants, coordination between leaf and root traits was explored [17]. Findings align with some studies but differ from tropical plant results, suggesting non-universal coordination [18]. Moreover, most research on plant economic traits is global, with limited focus on species levels with different life history community scales along environmental stress [19].

The riparian ecosystem serves as an ideal research site to investigate trade-offs between above- and below-ground traits as indicators of plant survival strategies in response to varying flooding intensities. Riparian zones, defined as ecotones influenced by floods at the interface of aquatic and terrestrial habitats, experience periodic water-level changes. This dynamic environment poses challenges for riparian communities, with studies indicating morphological variations among plant species along water-level gradients in response to flooding stress [20]. While roots are supposed to be crucial for submergence, leaf traits are the key to growth. Limited research exists on significant leaf-root trade-offs in riparian plants facing varying flooding intensities despite evidence suggesting adjustments in leaf morphology in high-flood environments to enhance survivability [21,22]. However, understanding trade-offs within riparian communities remains incomplete despite clues from resource allocation studies.

Hence, this study is motivated to identify the survival strategy of plants along a flooding intensity by examining the trade-offs between leaf and root traits at both community and species levels. The primary focus is on understanding how these traits respond to varying levels of flooding intensity and investigating potential adaptive strategies employed by riparian plants to cope with flooding stress. By conducting surveys and analyses of plant species within riparian communities, the study specifically addresses growth traits, physiological responses, and their correlations with soil factors. The research aims to address the following four questions: (1) Is there a correlation between leaf and root traits within the overall community in response to varying flooding intensity? (2) Are there significant trade-offs between leaf and root traits of individual dominant plants with different life histories under flooding stress? (3) Which soil factors play a pivotal role in regulating the trade-offs of plant traits along the stress gradient? (4) What potential strategies do the riparian plants employ along the flooding stress gradient?

## 2. Results

### 2.1. Coordination between Leaf and Root Traits of the Overall Community

Firstly, we tested the trade-offs of traits measured for all the dominant plants, rather than single species, within each sampled community. The dominant species include *Cynodon dactylon*, *Xanthium strumarium*, *Abutilon theophrasti*, *Phalaris arundinacea*, *Hemarthria sibirica*, *Cyperus michelianus*, *Persicaria lapathifolia*, *Melilotus officinalis*, *Alternanthera philoxeroides*, *Artemisia annua*, *Bidens pilosa*, *Bidens tripartita*, and *Cyperus rotundus.*

**Figure 1:**
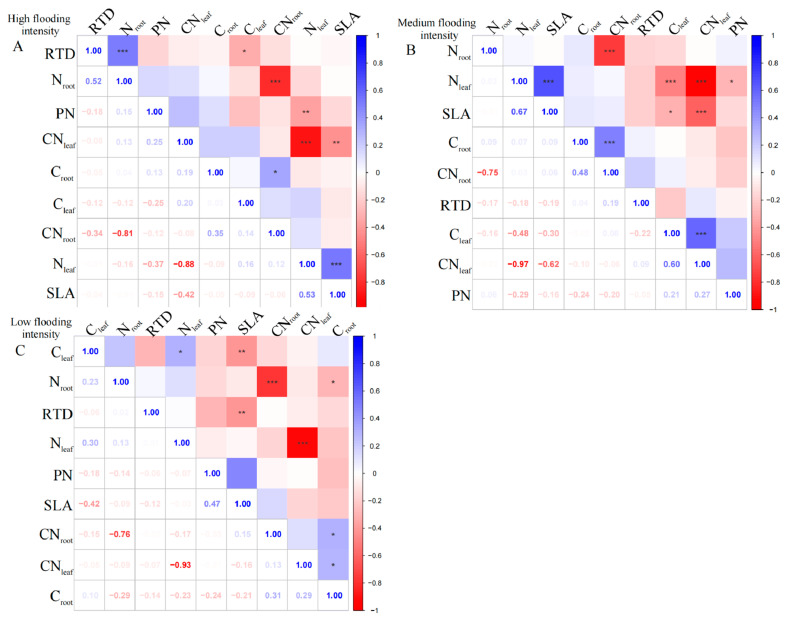
Correlation analysis and significant correlations for functional traits at different flooding gradients at the community scale. (**A**) Results of the analysis of high flooding intensity, (**B**) results of the analysis of medium flooding intensity, (**C**) results of the analysis of low flooding intensity. In correlation analysis, asterisk markers are used to indicate the significance level of the association between variables. * indicates *p* < 0.05, ** indicates *p* < 0.01, and *** indicates *p* < 0.001.

At the high flooding intensity, we only found a significant negative correlation (*p* < 0.01) between N_leaf_ and CN_leaf_ (Figure 1A). Furthermore, a highly significant positive correlation (*p* < 0.01) was observed between SLA and N_leaf_, indicating that a higher leaf area ratio corresponds to a higher N_leaf_. In addition, N_leaf_ showed a significant negative correlation (*p* < 0.01) with PN, indicating that higher nitrogen content in the leaves corresponded to a lower photosynthetic rate.

At the medium flooding intensity, CN_leaf_ was highly significantly negatively correlated with N_leaf_ (*p* < 0.01), indicating that there is a balance between nitrogen and carbon in plant leaves (Figure 1B). Furthermore, SLA was significantly correlated with C_leaf_, N_leaf_, and CN_leaf_ (*p* < 0.05), indicating that the nitrogen and carbon content of plant leaves and the carbon-to-nitrogen ratio have an important influence on photosynthesis and growth and development. However, RTD did not show a significant correlation with leaf and root nutrient ratios, indicating a weak relationship between RTD and plant nutrient uptake capacity.

At the low flooding intensity, N_leaf_ showed a significant positive correlation (*p* < 0.05) with C_leaf_, indicating a relationship between N_leaf_ and carbon content (Figure 1C). N_root_ was significantly negatively correlated with C_root_ (*p* < 0.05), indicating that plants under N deficient conditions use carbon for N uptake, supporting the previous results on N_leaf_ and CN_leaf_. SLA showed a highly significant negative correlation (*p* < 0.01) with C_leaf_, which may be related to the functional nature of leaves, which is related to the adaptation strategies. However, when assessing the overall community’s mixed species composition rather than single species, we found a statistically insignificant correlation between leaf and root nutrient traits across the intensity of flooding intensity.

### 2.2. Correlations of Leaf and Root Traits of Individual Dominant Species

To further test the coupling between leaf and root traits across the flooding gradient, three dominant species, *C. dactylon*, *A. theophrasti*, and *X. strumarium*, were selected and examined separately.

**Figure 2:**
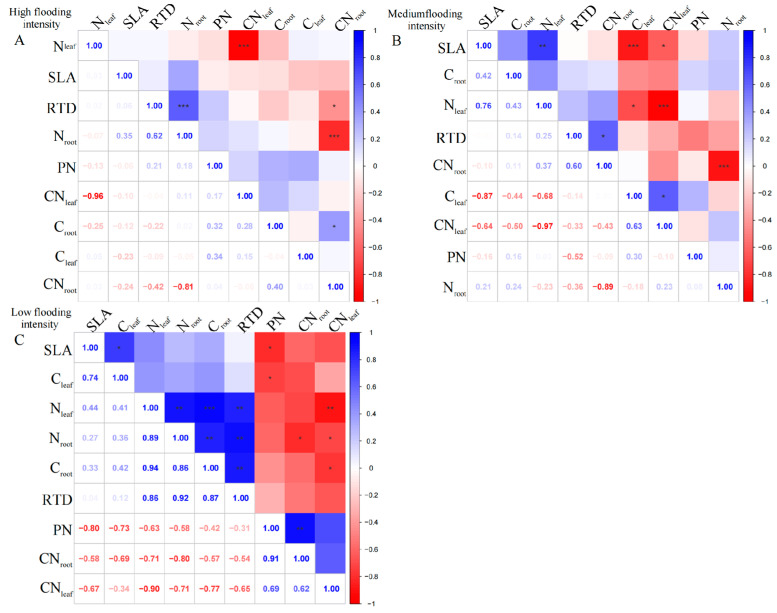
Correlation analysis and significant correlation of functional traits under different flooding gradients in *C. dactylon*. (**A**) Results of the analysis of high flooding intensity, (**B**) results of the analysis of medium flooding intensity, (**C**) results of the analysis of low flooding intensity.In correlation analysis, asterisk markers are used to indicate the significance level of the association between variables. * indicates *p* < 0.05, ** indicates *p* < 0.01, and *** indicates *p* < 0.001.

For the perennial species *C. dactylon*, our results showed complex linkages between different traits along the flooding gradient. At high flooding intensity, the results showed that N_leaf_ had a highly significant negative correlation with CN_leaf_ (*p* < 0.01); however, C_leaf_ was uncorrelated with N_leaf_ and CN_leaf_ (Figure 2A). In contrast, C_leaf_ and C_root_ in *C. dactylon* showed a weak correlation with the other indicators, probably because carbon content has less influence on plant growth and physiological characteristics. In addition, RTD showed highly significant positive correlations (*p* < 0.01) with both N_root_ and C_root_, indicating that roots with high N and carbon content may have higher water transfer efficiency. However, we found an insignificant correlation between leaf and root nutrients, which is consistent with the pattern under high flooding intensity at the entire community level.

At the medium flooding intensity, the results showed a significant negative correlation between N_leaf_ and C_leaf_ (*p* < 0.05), which may be related to the competition between nitrogen and carbon in the plant (Figure 2B). At the low flooding intensity, we found CN_leaf_ significantly correlated with C_root_ and N_root_, while N_leaf_ significantly correlated with C_root_, N_root_, and RTD (Figure 2C). This result suggests that there may be coordination between roots and leaves in terms of N use and allocation.

**Figure 3:**
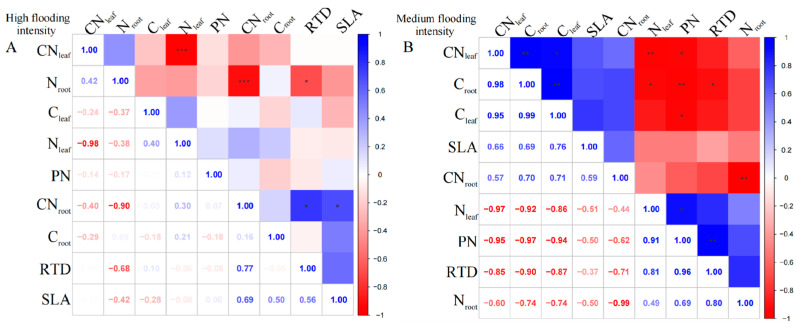
Correlation analysis and significance of different flooding gradients in *A. theophrasti*. (**A**) Results of the analysis of high flooding intensity, (**B**) results of the analysis of medium flooding intensity.In correlation analysis, asterisk markers are used to indicate the significance level of the association between variables. * indicates *p* < 0.05, ** indicates *p* < 0.01, and *** indicates *p* < 0.001.

For *A. theophrasti*, at the high flooding intensity, the results show that there was a significant negative correlation (*p* < 0.01) between N_leaf_ and CN_leaf_ (Figure 3A). Additionally, there was a significant negative correlation between N_root_ and CN_root_ (*p* < 0.01). CN_root_ also showed a correlation with SLA and RTD, respectively (*p* < 0.05), which indicates that CN_root_ may related to the growth and morphological characteristics of the species.

At the medium flooding intensity, C_root_ in *A. theophrasti* showed a significant negative correlation with N_leaf_ (*p* < 0.05), indicating that as C_leaf_ increased, N_leaf_ content decreased (Figure 3B). Also, C_root_ showed a highly significant positive correlation with C_leaf_ and CN_leaf_ (*p* < 0.01). Among the phenotypic traits, RTD was highly significantly positively correlated with PN (*p* < 0.01), indicating that leaf respiration rate was closely related to root tissue density, indicating that the leaf and root traits have links.

**Figure 4:**
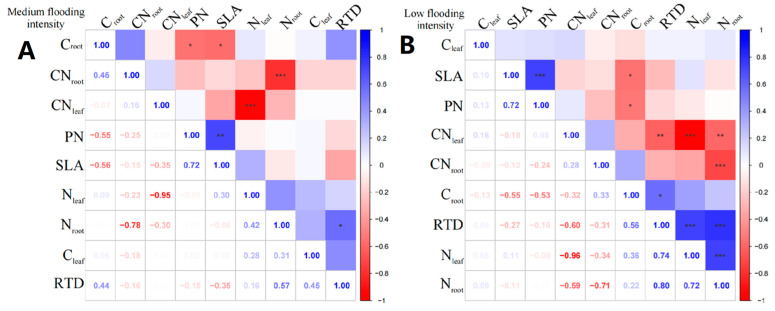
Correlation analysis and significance of different flooding gradients of *X. strumarium*. (**A**) Results of the analysis of medium flooding intensity, (**B**) results of the analysis of low flooding intensity. In correlation analysis, asterisk markers are used to indicate the significance level of the association between variables. * indicates *p* < 0.05, ** indicates *p* < 0.01, and *** indicates *p* < 0.001.

In *X. strumarium*, at the medium flooding intensity, N_leaf_ showed a positive correlation with C_leaf_ (Figure 4A), indicating that there is some coordination between nitrogen and carbon uptake and utilization during leaf development. C_root_ of *X. strumarium* showed a significant negative correlation with SLA and PN (*p* < 0.05), indicating that the lower the C_root_, the greater the SLA, and the greater the root load.

Under the low-intensity flooding condition, SLA showed a significant positive correlation (*p* < 0.05) with PN (Figure 4B). Additionally, RTD showed a significant negative correlation (*p* < 0.05) with CN_leaf_. In terms of linkages between leaf and root traits, C_root_ showed a significant negative correlation (*p* < 0.05) with SLA and PN.

### 2.3. Correlations between Soil Factors and the Traits Involved in Trade-Offs

In order to examine the key resource in shaping the trade-off traits, soil factors at the three levels of flooding intensity were tested for significant correlations with the trade-off traits plant significantly related traits, and traits with significant correlation were extracted.

**Figure 5:**
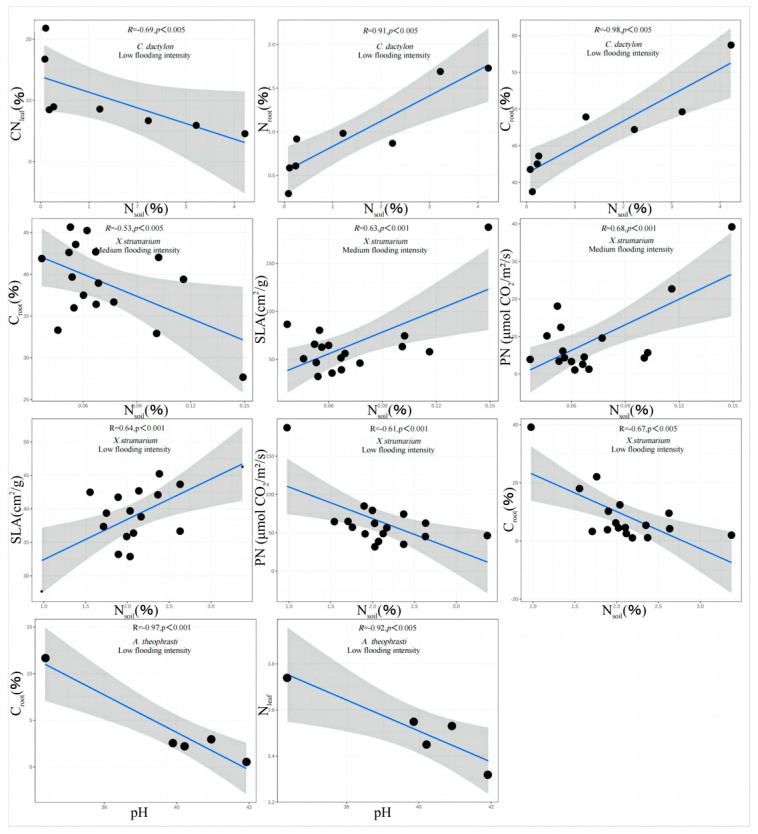
Correlation analysis and significance of different flooding intensity of soil.

**Figure 6:**
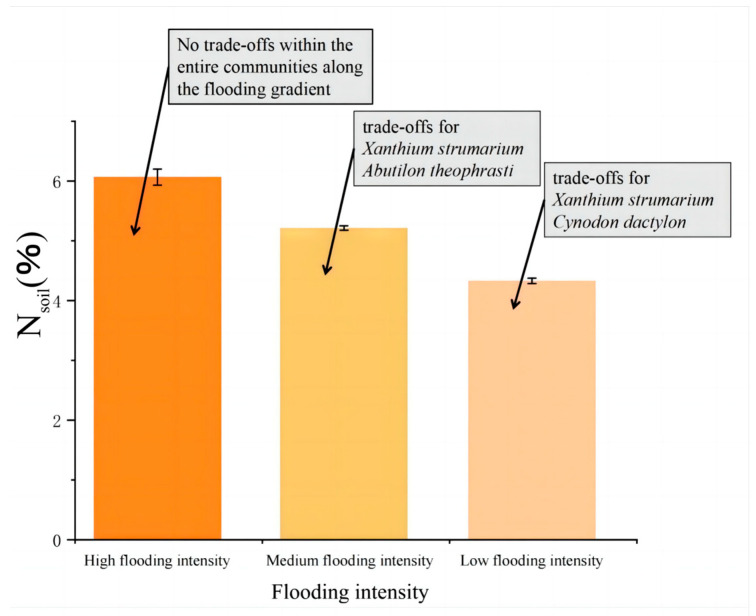
Trends in N_soil_. N_soil_ gradually decreased with the increase in flooding stress.

The results showed that there were correlations between N_soil_, pH, and the trade-off traits of *A. theophrasti*, *C. dactylon*, and *X. strumarium* along the flooding intensity (Figure 5). Specifically, significant correlations (*p* < 0.05) were observed for *C. dactylon* and *X. strumarium* with respect to N_soil_ at the low-intensity flooding, indicating that nitrogen availability in the soil is one of the important resources influencing the growth of *C. dactylon* and *X. strumarium*. *C. dactylon*, as a nitrogen-sensitive plant, exhibits restricted growth and development in response to changes in soil nitrogen levels. Similarly, *A. theophrasti*, which exhibits a similar response, showed a significant correlation (*p* < 0.05) between pH value and plant traits at the low flooding intensity. This suggests that soil pH plays a crucial role in the growth and performance of *A. theophrasti*. As a pH-sensitive plant, *A. theophrasti’s* physiological and ecological characteristics are closely related to soil pH levels. These findings highlight the importance of soil pH regulation in shaping plant fitness, particularly in environments with relatively low flooding intensity. N_soil_ decreases with increasing flooding intensity (Figure 6).

**Figure 7:**
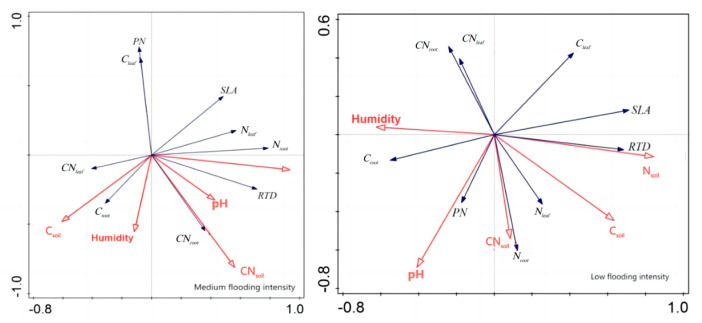
Redundancy analysis of soil and significant correlation traits of *X. strumarium*. Red color indicates soil factors, black color indicates plant functional traits.

**Figure 8:**
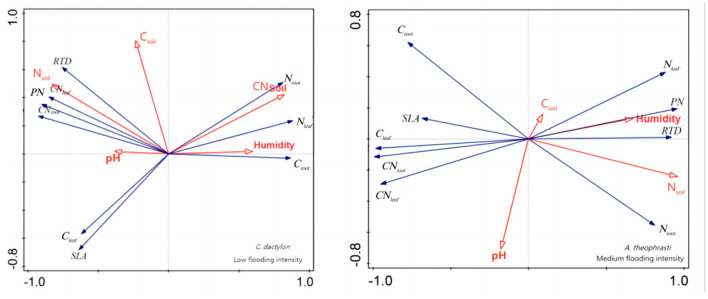
Redundancy analysis of soil and significant correlation traits of *C. dactylon* and *A. theophrasti*. Red color indicates soil factors, black color indicates plant functional traits.

In addition, redundancy analysis was performed on the soil factors and plant traits of *X. strumarium* (Figure 7). It showed that, under conditions of medium flooding intensity, N_soil_ was negatively correlated with C_root_ and positively correlated with pH and SLA. Notably, N_soil_ had the longest line length, indicating that this water level had the greatest impact on plant traits. At low flood intensity, N_soil_ was negatively correlated with PN C_root_ and positively correlated with SLA, which is the same as in the previous analysis. The redundancy analysis results of *C. dactylon* and *A. theophrasti* were also similar to the previous analysis results (Figure 8).

### 2.4. Niche Breadth of the Dominant Species

*C. dactylon*, as a perennial herbaceous plant, shows the characteristic of a generalist adapting across the intensity of flooding intensity (Table 1). It is worth noting that this species has a relatively wide niche breadth (0.763) at high flooding intensity. As the elevation increases at the medium flooding intensity, the niche breadth gradually decreases (0.627). This trend may indicate that *C. dactylon* has a specific adaptation or preference at high flooding intensity, while its competitive advantage may decrease at low flooding intensity.

On the contrary, *A. theophrasti* shows distinct patterns of ecological niche breadth. This species showed a significant habitat use (0.362) at high flooding intensity but decreased significantly (0.175) at medium flooding intensity. However, no individual was found at low flooding intensity. This variation may indicate that *A. theophrasti* has a specialism with a greater ability to adapt to more specific niches, which may be related to specific environmental conditions or resource availability.

Also, *X. strumarium* is categorized as an annual herbaceous plant. Notably, no individuals of the species were observed at high flooding intensity. However, a noticeable trend emerges with a widening habitat range as altitudes increase, particularly in response to moderate flooding intensity. This observation suggests that *X. strumarium* could be a specialist, demonstrating proficiency in coping with and adapting to a moderate level of flooding stress.

### 2.5. The CSR Model of the Main Dominant Species

**Figure 9:**
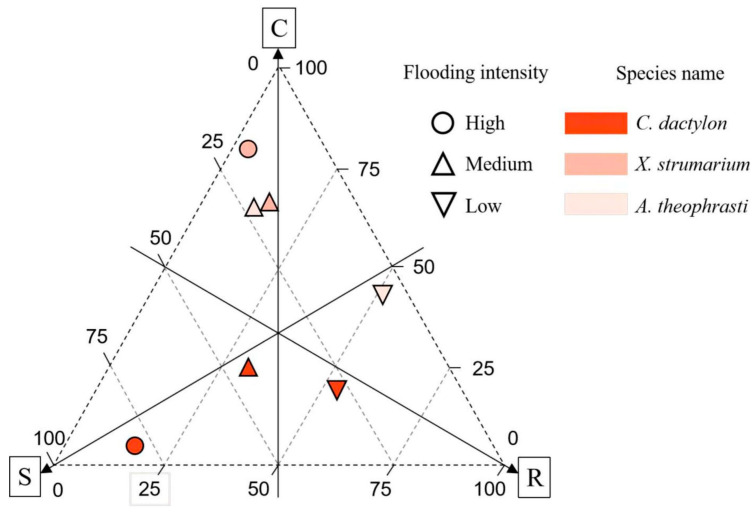
CSR Strategy of three dominant plants across flooding intensity. Note: C, Competitive; S, Stress-tolerant; R, Ruderal.

In the riparian communities, dominant plants exhibited a higher prevalence of the R-type strategy, followed by the S-type strategy, with the C-type strategy registering the lowest scores. Notably, *C. dactylon* displayed the lowest score for the R-type strategy. Different dominant plants manifest distinct adaptive strategies, with the perennial plant *C. dactylon* primarily relying on an S-type strategy. Furthermore, there is an observable increase in S-type strategy investment with a greater flooding intensity (Figure 9). The two annual species, *X. strumarium* and *A. theophrasti*, gradually switched from a C-type strategy to an R-type strategy with a decrease in flood intensity.

## 3. Discussion

### 3.1. No Trade-Offs of Leaf-Root Traits within the Entire Communities along the Flooding Intensity

This study comprehensively investigates the trade-offs between leaf and root traits of riparian communities along an intensity of flooding intensity. At the overall community level, rather than single species, we observed insignificant trade-offs across the three levels of flooding intensities. This phenomenon may indicate a diversity of adaptive strategies among different plant species at the community scale. Previous studies have emphasized that plants may adopt diverse adaptive strategies to ensure their survival and reproductive success when faced with different environmental pressures [23]. These diverse adaptive strategies may include aspects such as morphological traits, physiological mechanisms, and ecological niche distribution. Ecological niche distribution is the basis for species coexistence within plant communities and could be one of the major factors contributing to the diversity of plant adaptive strategies. Different plant species in the community may occupy different ecological niches, leading to differences in their adaptive strategies. Some species might be biased towards resource acquisition and growth, while others might prioritize survival in competitive environments [24,25]. Each plant species within the community may have different adaptive strategies, and this diversity may be a critical reason leading to the lack of clear trade-off strategies at the entire community scale. Therefore, the plant strategies cannot be indicated by the community-scale trade-offs of the relevant traits.

Further, the lack of clear trade-off strategies at the entire community level may be due to interactions and competitive relationships between different plant species. Competing and complementary relationships between plants may influence their trade-off behaviors. Additionally, environmental factors such as soil nutrients and water use efficiency may also influence the manifestation of plant trade-off strategies [26,27].

### 3.2. The Trade-Offs of Leaf-Root Traits Varied among Plant Species with Distinct Life Forms in Different Flooding Stress

In this study, the distinct trade-offs of leaf-root traits were observed for the single perennial and annual plants but showed different patterns. Our results indicate that perennial plants exhibit more pronounced trade-off strategies under low-intensity flooding, while annual plants exhibit significant trade-offs under both medium and low flooding intensity. The results showed that perennials exhibited a significant trade-off strategy under low-intensity flooding, while annuals exhibited a significant trade-off strategy under both medium and low-intensity flooding. The reason for this difference may be the long-term exposure of perennial plants to flood stress, as well as the fact that the riparian ecosystem is still in a stage of herbivorous succession.

The trade-off strategies of perennial plants are related to their life forms. Perennial plants thrive in water-rich environments, such as swamps and wetlands, and have longer life spans that require them to adapt to prolonged periods of flooding [28]. This chronic stress may lead perennial plants to evolve a number of adaptive mechanisms for resource allocation under flood conditions. Perennial and annual plants differ significantly in morphology and life cycle, which may lead them to adopt different trade-off strategies when faced with environmental stresses. Perennial plants have longer life spans and more complex root structures, which allow them to adapt to different environmental conditions and adopt different resource-use strategies. However, under a high flooding intensity, perennial plants may face more challenging resource allocation situations. Research suggests that under low flooding intensity, perennial plants may adjust their growth rates, reduce leaf area, or increase root branching to adapt to flood conditions [29].

In contrast, annual plants show significant trade-offs in resource allocation under both medium and low flooding intensity. This may be due to their niches requiring rapidly complete reproduction and life cycles within their short lifespans, leading to more rapid and efficient resource use. Annual plants often have fast growth rates, high reproductive capacities, and short lifespans, which allow them to rapidly utilize resources in the surrounding environment, including water [30]. Annual plants have short lifespans and simple life cycles and typically prioritize rapid growth and reproduction as their primary strategies [30]. Because they have shorter lifespans and do not have to endure prolonged flooding conditions, annual plants may have higher growth rates and greater biological adaptability to cope with changing flooding environments [31].

In addition, the environmental conditions of riparian zones also influence the resource allocation strategies of plants. The riparian zone is a dynamic transitional zone that is often affected by flooding and receding water. The unstable environmental conditions may require plants to balance resource use while adapting to water stress. Therefore, annual plants in the riparian zone may be more flexible in their resource allocation strategies to adapt to environmental changes [32]. Perennial plants exposed to long-term flood pressure adapt gradually and exhibit strong trade-off strategies, while annual plants exhibit greater flexibility and resource allocation strategies under different flood conditions.

The results emphasize the significant impact of flooding stress on plant resource allocation strategies and environmental adaptability. Annual and perennial plants adopt different resource allocation strategies under waterlogging stress to adapt to different levels of water logging. This adaptive strategy plays a crucial role in plant survival and reproduction in waterlogged environments [33]. Therefore, further study of plant resource allocation mechanisms and adaptive strategies is of great theoretical and practical value in understanding plant responses to waterlogging stress.

However, under high flooding intensity, no clear trade-off between leaves and roots was observed. SLA, a key characteristic in vegetation carbon sequestration strategies, is typically positively correlated with foliar nitrogen concentration per unit mass, leading to reduced carbon investment per unit leaf area and increased relative growth rates. Our results affirm a positive correlation between SLA and N_leaf_. However, the relationship between SRL and N_root_ is not significant (*p* > 0.05), aligning with findings in herbaceous plants. While previous studies have linked SRL to root nutrient uptake and lifespan, our results indicate a more complex relationship. Functional traits show certain correlations between leaves and fine roots, reflecting the holistic nature of plant growth metabolism. Existing research highlights a positive correlation between N concentration in leaves and root systems, but no consistent relationship between tissue morphology of leaves and fine roots was observed. The correlation between SLA and SRL is delicate and varies between species, consistent with previous research suggesting positive, negative, or unrelated relationships. Also, SLA and RTD exhibit inconsistent trends. A study has shown that the variation in leaf tissue density of 24 grassland plant species explained only 9% of the variation in fine root tissue density [34], while other studies have shown that there was no significant correlation between leaf and fine root tissue density for plants in four grassland regions worldwide [35]. Compared to leaf traits, plant root traits have greater variability and uncertainty [36], so the relationships between leaf and fine root traits are mainly influenced by fine root traits.

### 3.3. Soil Nitrogen Availability Determines the Trade-Offs of Leaf-Root Traits

The results of the study showed that there were significant correlations between the trade-off traits and N_soil_ across the flooding intensities. In particular, significant correlations (*p* < 0.05) were observed between N_soil_ and prominent plant traits in the soil of *C. dactylon* and *X. strumarium* at the medium and low flooding intensity. These findings are consistent with previous studies and support the importance of N_soil_ for the growth and adaptability of these plants [32,37].

As nitrogen-sensitive plants, the growth and development of *C. dactylon* and *X. strumarium* may be regulated by N_soil_. In high-nitrogen soils, these plants may have better access to and use of nitrogen resources, resulting in higher growth rates and biomass accumulation. However, in low-nitrogen soils, they may need to adapt to nitrogen-limited environments by adjusting root system structure, enhancing interactions with rhizosphere microorganisms, and optimizing metabolic pathways for nutrient uptake and utilization [38,39]. The regulation of such trade-off strategies may be crucial for plant survival and competitiveness under different soil conditions. Furthermore, we found that the N_soil_ of *C. dactylon* and *X. strumarium* plants decreased continuously. This suggests that under medium and low flooding intensity, N_soil_ decreases significantly, leading plants to balance different factors to compete for reduced soil nitrogen resources [40].

In addition, we also found a significant correlation (*p* < 0.05) between soil pH and significant plant traits of the plants under the medium flooding intensity *A. theophrasti*. This finding further supports the effect of soil pH on the growth and adaptability of *A. theophrasti*. Studies have shown that high soil pH can have an inhibitory effect on the growth of *A. theophrasti*, leading to reduced nutrient uptake and utilization capacity [41,42]. Therefore, in such environments, *A. theophrasti* may need to adjust its physiological and ecological traits, such as regulation of acid-base balance and secretion of acidic root exudates, to adapt to the challenges of high pH soils [43]. We observed a continuous increase in soil pH for *A. theophrasti*. This result suggests that degradation of the soil environment may prompt plants to adopt trade-off strategies to adapt to changing soil pH. Previous studies have already shown the detrimental effects of high soil pH on plant growth [44]. For example, high soil pH can lead to reduced nutrient uptake and utilization capacity in plants [45]. The results of our study are largely consistent with these findings and further emphasize the importance of soil pH for the ecological adaptability of plants under flood stress. Therefore, *A. theophrasti* may need to adopt compensatory strategies to cope with changes in soil pH. This may involve adjusting root morphology and structure to increase nutrient uptake capacity, regulating the interaction between rhizosphere microorganisms and plants to increase nutrient use efficiency, and modulating metabolic pathways to respond to environmental stresses.

### 3.4. Strategies of Dominant Plant Species along the Flooding Gradient

The combined utilization of niche breadth analysis and CSR model offers valuable insights into the adaptive strategies employed by the dominant species within the riparian communities. In this investigation, we constructed the CSR model by examining three key leaf functional traits. The results unveiled the adaptability of the dominant species along the flooding stress intensity concerning competition, stress, and disturbance. As flooding intensity diminishes, there is a gradual shift in the predominant adaptive strategy of plants toward the R-type. However, distinct distribution patterns of generalists and specialists were identified along the flooding stress gradient.

In this study, A noteworthy example is *C. dactylon*, a niche generalist and robust flood-tolerant species (S-type strategy), which adopts a conservative strategy under high flooding intensity. This perennial species demonstrates a robust rooting system without direct coordination with leaf traits, ensuring maximal resource utilization for survival in conditions of high flooding stress. Additionally, *C. dactylon* thrives in high-nitrogen soil, facilitating biomass accumulation. Further, as flood intensity decreased, the niche breadth of *C. dactylon* was found to decrease. This implies that even though the species possesses specific adaptations or tolerance for high flooding intensity, it might encounter diminished competitive advantages under low flooding intensity due to the rise in species diversity, which in turn intensifies competition for limited N_soil_. Given the global presence of *C. dactylon* [46], this identified strategy may represent a common model for this generalist and others in coping with environmental stress.

As a niche specialist, *A. theophrasti* is particularly evident in high flooding intensity. Meanwhile this species displays adaptive capability with an expanded niche breadth (0.362). On the other hand this species strong competitor employing a C-type strategy. However, its niche breadth experiences a rapid decline with increasing altitude, and this species was not recorded under low flooding intensity at the highest altitude range of 165–175 m. This variability underscores the adaptability of *A. theophrasti* to specific ecological fitness, potentially limited by specific environmental conditions or resource availability, which may relate to the declined N_soil_. These observations align with the CSR model of adaptive strategies, wherein C-type strategies dominate, emphasizing resource competition and utilization [5]. Conversely, *X. strumarium* exhibited a distinct pattern of niche breadth along the flooding gradient, indicating a typical R-type strategy. Although this species is also a specialist, it demonstrated an inability to thrive in high flooding intensity conditions. Examining the connections between leaf and root traits revealed noteworthy resource coordination.

By integrating insights from CSR strategy models, niche breadth analysis, and soil nitrogen content assessment, the results revealed that perennial species strategically adopt the S strategy in the face of higher flood pressures. This involves demonstrating resilience through conservative resource allocation to unlock leaf and root coordination. In contrast, annual species showed niche specialization across the flooding gradient, adopting different strategies, such as R- and C-strategies. With the decline of flood stress and the increase in soil nitrogen level, the plant strategy has a clear trend of transition to the R strategy.

This study provides valuable insights into the adaptive mechanisms of three globally distributed species, *C. dactylon*, *X. strumarium*, and *A. theophrasti*, in response to environmental stress. By examining the strategies employed by the three species, our comprehension of plant responses to environmental stress can be significantly enriched. In riparian ecosystems, soil nitrogen emerges as a pivotal regulator influencing plant growth, while flooding intensity serves as another critical factor affecting plant tolerance. Within the framework of these dual regulators, the manifestation of three distinct strategies (C-, S-, and R-type) becomes evident, aligning with Grime’s model. These strategies are characterized by trade-offs between leaf and root traits along the continuum of flooding intensity.

## 4. Materials and Methods

### 4.1. Study Area

This study was conducted on the riparian habitats along the Three Gorges Reservoir located at the Yangtze River in southern China. The Three Gorges Reservoir is located at the transition zone between the central subtropical and northern subtropical regions and is in the canyon area. Cold air is difficult to enter, and it is a typical warm winter area. Since 2009, the reservoir began to operate. According to the hydrological report over the years, the highest water level of 175 m in winter is about 16 days. The lowest water level of 145 m in summer is about 70 days, forming a large area of water fluctuation riparian zone.

The flooding stress created by the operation of the Three Gorges Reservoir has resulted in severe degradation of vegetation in the riparian zone and damage to the structure and function of the riparian ecosystem. The high inundation stress led to increased habitat fragmentation, rapid extinction of the original terrestrial vegetation, gradual succession of suitable vegetation, and significant changes in the vegetation pattern of the riparian ecosystem, the distribution of major plant species across the various altitudes in the riparian habitats along the Three Gorges Reservoir Area (Table 1) [47,48,49]. From the table, it can be seen that the main perennial plant in the riparian habitats is *C. dactylon*, and the main annual plants are *X. strumarium* and *A. theophrasti*.

Due to the influence of water flow and flood, the soil in the riparian area is mainly alluvial soil. Some sections of the middle and lower altitudes of the riparian area are mostly sand and gravel accumulation. The soil contains a large number of pebbles with poor water retention capacity and weak fertility. And the upper part is mainly composed of sandy loam soil, purple soil, lime soil, and loess.

### 4.2. Sampling Design

**Figure 10:**
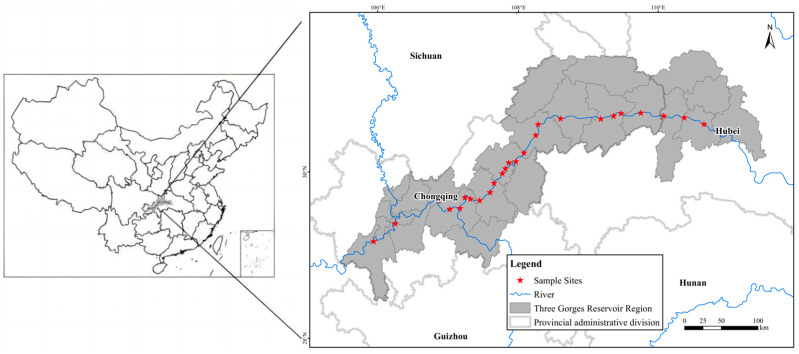
The study area and sampling sites along the Three Gorges Reservoir.

In this study, a total of 25 sampling sites on the riparian area across about 600 km of distance along the Three Gorges Reservoir area were selected (Figure 10).

**Figure 11:**
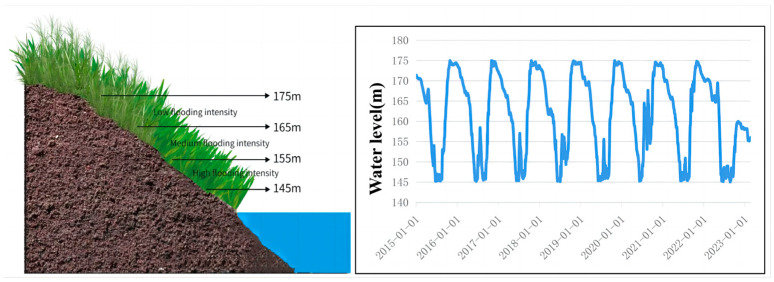
A schematic diagram of the sampling sites and water-level fluctuation.

In this study, we define the flooding intensity at altitudes 145–155 m as high level, at altitude 155–165 m as medium level, and at altitude 165–175 m as low level, along with other studies in the same area (Figure 11) [50]. A sample survey was conducted in July 2022 to collect plant samples once. At each sampling site, two sampling transects were constructed from the banks of the river edge to the upper slope. Within each sampling transect, two 1 × 1 m sampling quadrats were randomly placed at 5 m intervals (145–155–165–175 m) across the altitude. The schematic diagram shows daily water level fluctuations from 2016 to 2020, and three different levels of flooding intensity were chosen based on the flooding intensity formula [51]. The flooding intensity is calculated as FI = $\frac{Flooded\;days}{Whole\;days}$ for each level of flooding intensity, avoiding sparse areas during selection.

A total of 20 dominant species were recorded in this study. We chose *C. dactylon* as the focus of our perennial study because of its widespread occurrence in riparian areas and its prominence in many previous studies. In addition, we selected *A. theophrasti* and *X. strumarium* as the common annuals of interest. *A. theophrasti* and *X. strumarium* are the second most commonly observed annuals in the study area after *C. dactylon* [52,53], which allows them to be selected for the study (Table 2). On average, these three species collectively account for over 80% of the coverage within the riparian communities. Furthermore, these three species exhibit a global distribution and possess a robust ability to adapt to new habitats, frequently being found in areas that have undergone disturbance around the world.

Field surveys were conducted to record the species composition and the coverage of each species within each plant community. The nomenclature and species attributes follow the flora of China (http://www.iplant.cn/frps (Accessed July–September 2022)) and the local flora. Species richness was also recorded in each sampling quadrat. In addition, samples of the leaves and roots for each species in every quadrat were also collected.

### 4.3. Measurements of Leaf Traits, Root Traits and Soil Properties

For leaf collection, 3–5 leaves were collected from each plant, and the collected samples were put into an insulated box to be brought back to the laboratory. Then, the average leaf area, specific leaf area, leaf surface thickness, leaf dry matter content per unit mass, C_leaf_, and N_leaf_ were determined. Leaf surface thickness indicates water-holding capacity; leaf dry matter content and specific leaf area indicate relative growth rate and light-saturated photosynthesis rate; leaf N content indicates nutrient acquisition capacity and maximum photosynthetic rate. The photosynthesis rate and other indicators are measured by the HED-GH30 Plant Photosynthesis Measuring Instrument between 11:00 a.m. and 2:00 p.m., which ensures that the photosynthesis of the plant is at its highest. DM_leaf_ and SLA indicate relative growth rate and light-saturated photosynthetic rate, and N_leaf_ content indicates nutrient uptake capacity and maximum photosynthetic rate (Table S1).

We employed shovels or trowels to excavate the soil and expose the roots. The integrity of the roots was maintained in this process, avoiding damage or fracture, and the collected root samples were properly recorded and marked. Then, the samples were placed in a 0.05 mm mesh sieve for washing, and all roots were washed out. The roots were placed in an evaporator filled with distilled water, and the roots were separated from the impurities with tweezers. After being dried with absorbent paper, the Epson digital scanner was used to scan, and the root image analysis system software was used to determine the root length, root surface area, root volume, and root diameter. The root length density, root-specific surface area density, and root relative volume density were obtained by dividing the above three measurements by the root sampling volume. The roots, after morphological determination, were placed in an 80 °C oven and dried to constant weight to measure their dry weight.

The undisturbed soil of 0–20 cm depth was collected by profile excavation method. Three parallels were collected using a 100 cm3 ordinary ring knife sample for the determination of soil indicators. In order to eliminate the influence of season and soil moisture on soil, microbial soil samples and soil moisture samples were collected at the same time. The soil samples were sealed in a sterile self-sealing bag and quickly stored in an incubator filled with dry ice (−20 °C). After being brought back to the laboratory, they were stored in a refrigerator at −80 °C. Then, the basic physical and chemical measurements, such as pH, total carbon, and total nitrogen content in the soil, were determined (Table S1).

Finally, C, N, and P contents in roots were determined using an elemental analyzer (VarioTOC, Elementar, Germany) (Table S2). In this study, percentages (%) are employed as the measure of carbon or nitrogen content, with each percentage (1%) point representing 1 g of carbon or nitrogen content per 100 g of sample from leaves, roots, or soil. Thus, percentage measurements accurately reflect the real value of carbon or nitrogen content in the soil, roots, or leaves. For example, Moore analyzed the carbon, nitrogen, and phosphorus of Canadian forest litter decomposition in 2006 [54].

### 4.4. Niche Breadth

The extent of a species’ niche breadth serves as a valuable measure for discerning and comprehending its survival strategy. This metric offers insights into the spectrum of environmental conditions and resources that a species can effectively utilize or endure. Species endowed with a broad niche breadth may exhibit heightened resilience in challenging environments. Their capacity to exploit a diverse array of resources can confer a survival advantage, particularly in the face of stressors like flooding. Conversely, in stressful conditions, specialists characterized by a narrow niche may gain a survival edge. Their precise adaptations enable more effective coping with stress factors. The relationship between species niche breadth and survival strategy is intricate and contingent on the specific ecological context. It necessitates a delicate balance between adaptability and efficiency, where the ecological circumstances determine whether a broad or narrow niche breadth is more advantageous for survival.

In this study, the ecological niche breadth is calculated using the following formula proposed by Corwell [55]:

(1) $$Bi=\frac{1}{r\times \sum_{h=1}^{r}p_{ih}^{2}}$$

where *Bi* is the ecological niche width of the *i*-th species; *p_ih_* is the proportion of the importance value of the *i*-th species at the *h*-th resource level to the total importance value of the species at all resource levels; *r* is the level of resources; the value range of the formula is [0, 1].

### 4.5. CSR Model

Grime’s CSR model is a widely used ecological framework that categorizes plant species into three strategies: Competitors (C), Stress-tolerators (S), and Ruderals (R). Competitors thrive in stable environments with abundant resources, stress-tolerators endure adverse conditions, and ruderals excel in disturbed environments with rapid growth and reproduction. The model highlights trade-offs among these strategies, providing valuable insights into how plants adapt to varying ecological niches and environmental challenges.

Using the results obtained from the primary dominant plants, we established the CSR model. This approach delineates the ecological adaptation strategy of the plants by examining the quantitative relationship among three leaf functional traits: specific leaf area (SLA), which serves as an indicator of the plant’s adaptability to competition, stress, and interference gradients [56].

### 4.6. Statistical Methods

The data were analyzed using IBM SPSS Statistics 24.0 for descriptive statistics and R Statistical Software (version 4.2.3) with R Studio (version 2022.02.2) for graphical representation and significance calculations. Univariate statistics, including means, standard deviations, minimums, and maximums, were computed in SPSS to assess data distribution and identify outliers. The normality of continuous variables was evaluated using histograms and the Shapiro-Wilk test, and Levene’s test checked the homogeneity of variance. Data transformations, such as logarithmic or square root, were applied as needed to meet assumptions.

Ordination methods were employed to assess coordination between leaf and root traits along flooding stress gradients. Redundancy analysis, a form of constrained ordination, was used to identify explanatory variables affecting trait changes. The method visually represented the relationship between plant traits and flooding conditions, with arrow length indicating the degree of correlation and angles representing positive or negative correlations.

Furthermore, one-way ANOVA tests were conducted in SPSS to analyze differences in mean values across categorical groups, with Tukey’s post hoc tests for pairwise comparisons. *T*-tests were used for comparisons between two groups, and non-parametric tests (Kruskal–Wallis, Mann–Whitney U) were applied if assumptions were violated. All analyses were two-tailed at a significance level of 0.05, with *p*-values reported unless very small (*p* < 0.001). Descriptive statistics were presented as mean ± standard deviation or median (interquartile range), and ggplot2 in R (version 4.2.3) was used for graphical representation.

## 5. Conclusions

In this study, our primary objective was to investigate how plant species within riparian communities manage trade-offs between leaf and root traits across a flooding intensity gradient. Through the observation and analysis of the co-variant responses of these traits to varying flooding stress, we drew significant conclusions as below:

1. The overall riparian communities did not display consistent trade-offs along the flooding intensity gradient. This lack of consistency may arise from individual species within the community employing diverse adaptive strategies, resulting in the absence of uniform trade-off patterns within the same community;
2. Noteworthy trade-off differences between perennial and annual plants were observed, indicating that distinct plant morphologies drive diverse resource use strategies and adaptability. The trade-offs were evident in traits related to nitrogen-carbon resources. This underscores the evolutionary dynamics of trade-off strategies for these plant types, emphasizing that strategies may be specific to individual species with different life histories rather than reflective of the overall community;
3. However, under strong flooding conditions, all studied species exhibited unclear trade-offs, suggesting inherent trait fitness favoring a more conservative strategy. This fitness enabled these plants to concentrate functionally on root traits under high flooding intensity, contradicting optimized trade-offs observed in low and moderate flooding conditions;
4. Soil nitrogen in the riparian zone emerged as a crucial regulator for leaf-root trade-offs under moderate and low flooding intensity. Significant correlations were established between soil nitrogen and traits involved in trade-offs, highlighting the regulatory role of soil nitrogen in riparian plant resource economics when flooding stress is not pronounced;
5. Through the integration of CSR model analysis and the evaluation of niche breadth, we noted a transition in the primary adaptive strategy of plants from C-type and S-type to R-type as flooding intensity decreased. This shift was accompanied by a convergence in niche occupation between generalist and specialist species. Influenced by the dual factors of soil nitrogen and flooding stress, the emergence of all three distinct strategies (C-, S-, and R-type) highlighted the balance between the growth and tolerance of riparian plants, which is characterized by trade-offs in leaf and root traits along the intensity of flooding intensity.

Forthcoming research ought to concentrate on revealing the mechanisms behind the formation, the thresholds for transformation, and the ranges of fluctuation in dynamic adaptive trade-off strategies within riparian plants.

## Figures and Tables

**Table 1:** Changes in niche breadth of three dominant species along flooding intensity.

Species Number	Species Name	Lifestyle	Flooding Intensity (FI)		
			High	Medium	Low
1	*C. dactylon*	Perennial herb	0.763	0.527	0.152
2	*A. theophrasti*	Annual herb	0.362	0.175	-
3	*X. strumarium*	Annual herb	-	0.426	0.364

**Table 2:** Distribution of the major plant species in the study area.

Vegetation Type	Altitude		
	145–155 m High Flooding Intensity	155–165 m Medium Flooding Intensity	165–175 m Low Flooding Intensity
Annual plant	*Polygonum hydropiper*	*Symphyotrichum subulatum*	*Erigeron annuus*
	*Phyllanthus ussuriensis*	*Aster tataricus*	*Cyperus microiria*
	*Xanthium strumarium*	*Erigeron sumatrensis*	*Torenia violacea*
	*Polygonum orientale*	*Phyllanthus ussuriensis*	*Amphicarpaea edgeworthii*
	*Ageratum conyzoides*	*Pouzolzia zeylanica*	*Lagedium sibiricum*
	*Ipomoea aquatica*	*Digitaria chrysoblephara*	*Artemisia selengensis*
	*Benincasa hispida*	*Setaria viridis*	*Cnidium monnieri*
	*Gnaphalium affine*	*Abutilon theophrasti*	*Xanthium strumarium*
	*Abutilon theophrasti*	*Bidens pilosa*	*Bidens tripartite*
		*Xanthium strumarium*	*Gnaphalium affine*
Perennial plant	*Cyperus rotundus*	*Alternanthera philoxeroides*	*Dysphania ambrosioides*
	*Cynodon dactylon*	*Pilea subcoriacea*	*Senecio scandens*
	*Echinochloa oryzoides*	*Mazus miquelii*	*Humulus scandens*
		*Oplismenus undulatifolius*	*Vicia hirsuta*
		*Cynodon dactylon*	*Clinopodium chinense*
			*Cynodon dactylon*

## Data Availability

Source data are available from the authors upon request.

## Supplementary Materials

The following supporting information can be downloaded at https://www.mdpi.com/article/10.3390/plants13070978/s1, Table S1: Leaf and root traits, as well as soil factors measured in this study. Table S2: The mean values of plant traits at community scale, *C. dactylon*, *X. strumarium* and *A. theophrasti* under different flooding intensity.
