# Supplementary material for: Soil Nitrogen and Flooding Intensity Determine the Trade-Off between Leaf and Root Traits of Riparian Plant Species
Source: Plants (Basel). 2024 Mar 29;13(7):978. doi: 10.3390/plants13070978 (PMC11013260; doi:10.3390/plants13070978)
Supplement: Supplementary file 1 [file plants-13-00978-s001.zip › plants-2874112-supplementary.pdf]

Supplementary Material

Table S1 Leaf and root traits, as well as soil factors measured in this study.

	Parameter name	Abridge
Leaf	Specific leaf area (cm^2/g)	SLA
	Leaf dry matter content (g/g)	DM_{leaf}
	Leaf C content (%)	C_{leaf}
	Leaf N content (%)	N_{leaf}
	Leaf Carbon to Nitrogen Ratio	CN_{leaf}
	Photosynthetic rate (μmol $CO_2/m^2/s$)	PN
Root	Root tissue density (g/cm^3)	RTD
	Root C content (%)	C_{root}
	Root N content (%)	N_{root}
	Root Carbon to Nitrogen Ratio	CN_{root}
Soil	Potential of hydrogen	pH
	Soil C content (%)	C_{soil}
	Soil N content (%)	N_{soil}
	Soil Carbon to Nitrogen Ratio	CN_{soil}
	Soil Moisture Content (%)	Humidity

Table S2 The mean values of plant traits at community scale, *C.dactylon*, *X.strumarium* and *A.theophrasti* under different flooding intensity.

	Flooding intensity (FI)	N_{leaf} (%)	C_{leaf} (%)	CN_{leaf}	N_{root} (%)	C_{root} (%)	CN_{root}	PN (μmol $CO_2/m^2/s$)	SLA (%)	RTD (%)	N_{soil} (%)	C_{soil} (%)	CN_{soil}	pH	Humidity
Community	High flooding intensity	2.57	39.46	16.47	0.92	37.95	46.39	15.08	6.11	13.04	0.13	1.95	22.92	8.21	32.21
	Medium flooding intensity	2.93	39.35	14.32	0.79	37.31	51.13	13.13	33.78	1.45	0.10	1.94	18.62	8.13	29.73
	Low flooding intensity	3.19	40.18	13.52	0.64	38.34	73.64	30.48	33.56	1.28	0.08	2.01	22.48	7.72	25.83

Cynodon dactylon	High flooding intensity	2.28	40.88	18.59	0.92	38.58	46.19	15.22	1.33	2.12	0.14	2.00	23.68	8.15	30.16
	Medium flooding intensity	2.30	40.19	18.23	0.79	36.36	49.15	23.35	2.20	1.65	0.11	2.14	21.76	8.17	26.31
	Low flooding intensity	4.99	40.18	10.21	0.96	46.39	48.32	7.83	4.81	0.88	1.44	5.21	20.59	8.04	32.88
Abutilon theophrasti	High flooding intensity	4.91	39.27	8.17	0.86	37.44	47.54	6.54	79.22	1.36	0.10	1.97	19.99	8.11	28.66
	Medium flooding intensity	3.52	38.77	11.05	0.80	39.86	52.36	1.58	74.82	3.98	0.13	2.31	18.11	8.24	26.78
Xanthium strumarium	Medium flooding intensity	3.46	38.36	11.19	0.84	39.00	52.60	8.96	64.72	0.84	2.09	0.17	17.85	8.22	31.51
	Low flooding intensity	4.27	39.96	9.36	1.64	46.29	28.22	2.15	46.71	3.08	1.11	3.37	3.03	8.60	37.39
